# Visceral Leishmaniasis Revealing Undiagnosed Inborn Errors of Immunity

**DOI:** 10.1590/0037-8682-0322-2023

**Published:** 2023-11-10

**Authors:** Daniel Gleison Carvalho, Dewton de Moraes Vasconcelos, Andreia Cristiane Rangel Santos, Jose Angelo Lauletta Lindoso

**Affiliations:** 1 Instituto de Infectologia Emílio Ribas, São Paulo, Brasil.; 2 Universidade de São Paulo, Faculdade de Medicina, Hospital das Clínicas, Laboratório de Investigação Médica em Dermatologia e Imunodeficiência, São Paulo, Brasil.; 3 Universidade de São Paulo, Faculdade de Medicina, Hospital das Clínicas, Laboratório de Investigação Médica em Pediatria, São Paulo, Brasil.; 4 Universidade de São Paulo, Faculdade de Medicina, Hospital das Clínicas, Laboratório de Investigação Médica em Protozoologia, Bacteriologia e Resistência Antimicrobiana, São Paulo, Brasil.

**Keywords:** Visceral Leishmaniasis, Primary Immunodeficiency, Cellular immunodeficiency

## Abstract

Visceral Leishmaniasis (VL) is a potentially fatal disease and may be associated with primary or acquired immunodeficiencies. There are few reports, in the literature, of inborn errors of immunity. Here, we report two cases of VL as a marker of inborn errors of immunity, namely, GATA2 and RAB27A deficiency. Our data suggest that VL patients should be screened for primary immunodeficiency, particularly in cases of VL relapse.

## INTRODUCTION

Visceral Leishmaniasis (VL) is caused by protozoa of the genus *Leishmania* and transmitted by sandflies. Clinically, VL is characterized by fever, pancytopenia, weight loss and hepatosplenomegaly, but it can be asymptomatic^1^. The incidence of VL has, increased in immunosuppressed patients, mainly people living with HIV/AIDS^2^, but VL has been reported in immunodeficient patients, mainly in organ transplants and immunosuppressant drug treatment^3^. Despite the increased incidence of VL in immunosuppressed patients, its association with primary immunodeficiencies is almost unreported. Some case reports have shown that, in several primary immunodeficiencies VL may be the first manifestation of immunodeficiency with an unusual clinical manifestation or as a disease refractory to treatment^4^ ^,^ ^5^ ^,^ ^6^. However, there is a lack of knowledge about the occurrence of VL in patients with inborn errors of immunity (IEI), or a lack of investigation of primary immunodeficiency in patients with unusual clinical forms or refractory to treatment. Here, we report two cases of VL as markers of undiagnosed primary immunodeficiencies, because the diagnosis of VL triggered the investigation and diagnosis of an IEI. No previous reports were found in the specialized literature (Cochrane Library, LILACS, SciELO, MEDLINE, PubMed, and PMC (PubMed Central).

## CASE REPORT 1

Male, 37 years old, born in Maranhão (Northeast Brazil), sugarcane cutter, son of consanguineous marriage and occasional drinker. At the age of 21, he presented with inflammatory arthralgia affecting the elbows, wrists, hands, knees and ankles. Rheumatoid arthritis was diagnosed and treatment with non-steroidal anti-inflammatory drugs and prednisone commenced. There was improvement in symptoms, with maintenance of corticosteroid therapy (10 mg/day of prednisone) for 7 years. He and his family did not have a history of recurrent infections. He was admitted to another hospital with a history of daily fever, up to 39°C, for 5 months, asthenia, weight loss of approximately 10 kg and increased abdominal volume. Physical examination revealed pale skin and mucous membranes and splenomegaly (21 cm below the left costal margin) and hepatomegaly (4 cm from the right costal margin). Laboratory tests showed pancytopenia, in addition to hypoalbuminemia (Table 1). The bone marrow aspirate showed relative hypocellularity of the granulocytes, but without visualization of amastigotes of *Leishmania*. Bone marrow biopsy showed a proportional increase in granulocytes. Liver biopsy was also performed, with immunohistochemistry negative for *Leishmania*. ELISA immunoassay and rK39 rapid test was positive. Polymerase chain reaction (PCR) was performed on the bone marrow aspirate, with a positive result for *Leishmania*. Treatment with liposomal amphotericin B (LAB) at a total cumulative dose of 40mg/kg, for 10 days. An HIV test for further immunodeficiencies was negative. Peripheral blood immunophenotyping revealed a CD4+ lymphocyte count of 154 cells/mm³ (normal range: 493-1666 cells/mm³), absence of B lymphocytes (normal range: 72-520 cells/mm³), 8 NK cells/mm3 (normal range: 73-654 cells/mm³) after the first VL treatment. GATA2 deficiency was detected by dideoxynucleotide sequencing. Due to unresponsive treatment to VL, interferon gamma associated with LAB and meglumine antimoniate at a dose of 10mg/kg, for 16 days (suspended on the 16th day after increasing the QTc interval). Additionally, empirical treatment for *Mycobacterium avium* complex (MAC) with rifabutin, clarithromycin and ethambutol was initiated.

**TABLE 1:** Clinical and laboratory abnormalities described in the two cases reported, upon diagnosis of visceral leishmaniasis.

	Patient 1 (GATA2 deficiency)	Patient 2 (Griscelli syndrome)
Spleen size	21 cm below the left costal margin	23 cm below the left costal margin
Liver size	4 cm from the right costal margin	5 cm from the right costal margin
Hemoglobin	5.7 g/dL	10.9 g/dL
White blood cells count	1200 cells/mm^3^	6900 cells/ mm^3^
Neutrophils count	900 cells/ mm^3^	1600 cells/ mm^3^
Lymphocytes count	100 cells mm^3^	5000 cells/ mm^3^
Platelets count	< 10,000/ mm^3^	130,000/ mm^3^
Creatinine	0.57 mg/dL	0.73 mg/dL
Urea	30 mg/dL	21 mg/dL
Total Bilirubin /direct fraction/indirect fraction	1.44/ 1.13/ 0.31 mg/dL	0.47/ 0.11/ 0.36 mg/dL
Gamma-glutamyl transferase	420 U/L	56 U/L
Alkaline phosphatase	62 U/L	471 U/L
Lactic desidrogenasis	734 U/L	619 U/L
Aspartate aminotransferase / alanine aminotransferase	51/123 U/L	41/16 U/L
Albumin/globulins	2.4 /2.7 g/dL	4.5/2.8 g/dL

The patient showed clinical improvement after 5 days of combined treatment for VL and was discharged in good health. We observed resolution of splenomegaly and pancytopenia after 5 months of follow-up. The patient was referred for hematopoietic stem cell transplantation at another institution.

## CASE REPORT 2

**FIGURE 1:**
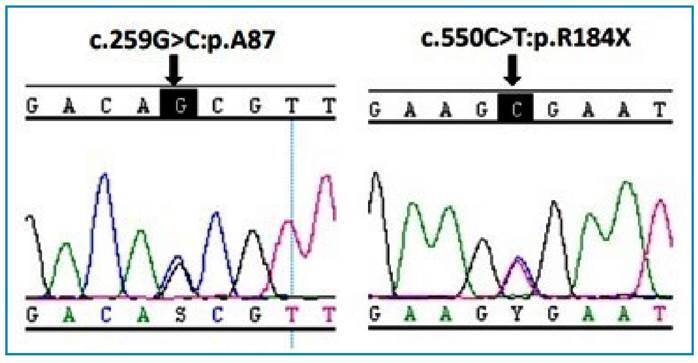
Electropherogram of RAB27A gene sequencing showing the compound heterozygous mutation.

A 13-year-old boy, born in São Paulo, with a history of traveling at age 12 to the state of Minas Gerais (Southeast Brazil). He had fever on alternate days, pallor, increased abdominal volume and unquantified weight loss. He did not have a history of recurrent infections. Anemia and thrombocytopenia were found on admission (Table 1). Bone marrow aspirate showed amastigotes of *Leishmania*. He received treatment with LAB at a dose of 35 mg/kg. Four months after hospital discharge, he again presented with fever and thrombocytopenia at the outpatient clinic. A bone marrow aspirate was performed, showing amastigotes of *Leishmania* that were also detected in his bone marrow culture. He was treated with meglumine antimoniate and intravenous human immunoglobulin, with clinical and laboratory improvement, and was discharged in good health. Three weeks later, with a seven-day history of fever, weakness, pallor and increased abdominal volume, a palpable spleen was detected 23 cm below the left costal margin and a palpable liver 5 cm from the right costal margin. *Leishmania* ELISA immunoassay and the rK-39 antigen test were negative. Bone marrow biopsy showed a lymphocytic and histiocytic inflammatory reaction with a granulomatous pattern; no amastigotes were observed in the bone marrow aspirate. He was re-treated for VL, on an empirical basis, with LAB. The patient had a phenotype suggestive of Griscelli Syndrome (silvery hair and eyebrows). Next-generation sequencing (NGS) was performed for a panel of genes associated with familial hemophagocytic lymphohistiocytosis, which showed a compound heterozygous mutation in the RAB27A gene (Figure 1), diagnostic of Griscelli Syndrome type II. He received LAB every 15 days as secondary prophylaxis for 6 months, with follow-up at an immunology service at another institution.

## DISCUSSION

The protective immune response against species of the genus *Leishmania* is essentially cell-mediated. Environmental and host factors also contribute to greater susceptibility to the disease^7^. Primary immunodeficiencies (PIDs), also known as IEI, are characterized by alterations in the functioning of the immune system, corresponding to several conditions, which may be congenital or manifest later in life^8^. Clinically, most cases of IEI are highly susceptible to common infections. Early diagnosis of IEI is important to initiate appropriate management, however up to 40% of cases are diagnosed only in adulthood^8^.

GATA-binding protein 2 (GATA2) is one of six GATA-binding factors that act to regulate gene expression for hematopoiesis, allowing hematopoietic stem cells to survive and self-renew. GATA2 deficiency can occur due to gene deletion, mutations in regulatory regions or exonic variations of nucleotides^9^. Mutation carriers are hematologically normal at birth, with hematological phenotypes manifesting over time, and mononuclear cell loss appears to be common to all symptomatic patients. Patients and families with documented disabilities require genetic counseling. Median survival is approximately 60 years and patients can be periodically followed up with leukocyte count and myelogram annually^9^.

Infections in patients with GATA2 mutations are predominantly secondary to dysfunction of cell-mediated immunity, with monocytopenia, which leads to profound dysfunction of the cytokine response, absence of IFNγ/IL-12 response, predisposing to atypical mycobacterial infections (in 20-50% of individuals), aspergillosis (16%), histoplasmosis (9%) and warts by all HPV serotypes (60-70%)^9^ ^,^ ^10^. Our patient with GATA2 deficiency was empirically treated for atypical mycobacterial infection. Importantly, he was diagnosed with VL, which has never been reported in individuals with this underlying immunodeficiency. We strongly suggest that the GATA 2 mutation was the trigger for the development of VL, because of which the infection, in the context of IEI, had an unfavorable evolution, with relapses after treatment, requiring secondary prophylaxis.

Griscelli Syndrome (GS) is commonly diagnosed between 4 months and 7 years of age, unlike our reported patient, with diagnosis made in adolescence. In GS type II, changes in skin and hair color occur, associated with immunological abnormalities and the development of hemophagocytic lymphohistiocytosis (HLH)^11^. The RAB27A gene (mutated in GS type 2) is expressed in leukocytes, melanocytes and other cell types encoding a RabGTPase that is responsible for vesicular transport in melanocytes (melanosomes), cytotoxic T lymphocytes and NK cells (cytolytic granules)^12^. Lytic granules contain perforin and granzyme, which induce apoptosis of infected cells, in addition to histiocyte activation and hypercytokinemia. Immunodeficiency in GS affecting lymphocytes is one of the factors that may predispose the patient to develop VL. Diagnosis should be as early as possible, as individuals are predisposed to recurrent infections and HLH, which can be fatal. Hematopoietic stem cell transplantation is the only treatment for this condition^12^. Our reported patient had the characteristic phenotype of GS, but no history of previous recurrent infections, the diagnosis being made only after recurrence of VL. To our knowledge, this is the first reported case of VL in a patient with GS. As both GATA 2 deficiency and Griscelli syndrome affect the cellular response, facilitating the infection of intracellular and intravesicular pathogens, such as mycobacteria and fungi, we assume that protozoa exhibit opportunistic behavior in patients with these immunodeficiencies. Therefore, it is expected that patients with immunosuppression will present a higher parasite load, however in case report 1, no amastigote forms were found in bone marrow, as described in case report 2. We can assume in this case, the parasites would reside in other organs, such as the spleen or gastrointestinal tract, however we cannot confirm this hypothesis, as there was no evaluation of these organs.

According to our data, we strongly suggest that VL manifested itself as an opportunistic disease in the two reported cases, as it’s known that control of *Leishmania* infection depends on an effective cellular response, which does not occur in patients with Griscelli syndrome or with GATA 2 mutations. 

To our knowledge, these are the first reported cases of an association between VL and GATA2 deficiency and Griscelli syndrome. 
